# Integrated single-cell and experimental analyses implicate GPRC5B in macrophage senescence-related alterations in postmenopausal osteoporosis

**DOI:** 10.3389/fimmu.2026.1890278

**Published:** 2026-08-18

**Authors:** Shizhou Zhao, Yixin Yang, Jiao Meng, Zhenkun Yang, Xinyi Jiang

**Affiliations:** 1 Department of Orthopedics, The Affiliated Wuxi People’s Hospital of Nanjing Medical University, Wuxi People’s Hospital, Wuxi Medical Center, Nanjing Medical University, Wuxi, China; 2 Department of Microbiology and Immunology, Yong Loo Lin School of Medicine, National University of Singapore, Singapore, Singapore; 3 Department of Laboratory Medicine, The Affiliated Wuxi People’s Hospital of Nanjing Medical University, Wuxi People’s Hospital, Wuxi Medical Center, Nanjing Medical University, Wuxi, China

**Keywords:** bone marrow microenvironment, DLG1/Kv1.3 axis, GPRC5B, macrophage dysfunction, post-menopausal osteoporosis, senescence-associated changes

## Abstract

**Background:**

Post-menopausal osteoporosis (PMO) is characterized by senescence, estrogen deficiency, immune dysregulation and progressive bone loss, yet the molecular alterations involved in PMO remain incompletely understood.

**Methods:**

Single-cell and transcriptomic analyses were integrated with machine learning and experimental validation. Ovariectomized mouse models, primary bone marrow-derived macrophages and RAW264.7 cells were used to evaluate gene expression, senescence-associated changes, macrophage polarization and related signaling pathways.

**Results:**

Single-cell RNA sequencing revealed substantial remodeling of the bone marrow microenvironment in PMO and identified APOE-positive macrophages as a dominant subset with elevated KRAS-related signaling. Integrated co-expression and machine-learning analyses yielded GPRC5B as a PMO-associated candidate gene that was consistently upregulated across multiple datasets. *In vivo* and *in vitro*, GPRC5B knockdown attenuated senescence-associated changes in macrophages, reduced inflammatory activation, promoted M2-like features and improved phagocytic clearance of apoptotic osteoblasts. Mechanistically, GPRC5B was associated with DLG1/Kv1.3-related signaling, and rescue experiments supported the involvement of this axis in the macrophage phenotypes. In PMO mice, GPRC5B knockdown was accompanied by apparent trend toward improved bone structure, and this effect was enhanced by DLG1 inhibition. Drug-signature screening and docking analysis further identified Doramectin as a candidate compound, whose treatment was associated with reduced GPRC5B expression under estrogen-deprived conditions.

**Conclusions:**

GPRC5B is a macrophage-enriched, PMO-associated candidate identified through integrated transcriptomic analyses and supported by preliminary functional experiments. These findings suggest that GPRC5B may participate in macrophage-related alterations in PMO and warrant further mechanistic and translational investigation.

## Introduction

Post-menopausal osteoporosis (PMO) is one of the most common metabolic bone disorders in elderly women and is characterized by reduced bone mass, deterioration of bone microarchitecture, and increased fragility fracture risk (1, 2). With increasing life expectancy worldwide, PMO has become a major health burden because osteoporotic fractures not only impair mobility and quality of life, but also contribute substantially to disability, hospitalization, and mortality in aging populations (3). Although anti-resorptive and anabolic therapies have improved clinical management, a considerable proportion of patients still experience progressive bone loss, limited long-term benefit, or treatment-related adverse effects (4). These limitations underscore the need to better define the molecular and cellular mechanisms underlying PMO and to identify more effective therapeutic targets.

PMO has long been viewed primarily as a consequence of estrogen deficiency. However, growing evidence indicates that the pathogenesis of PMO extends beyond endocrine imbalance and involves profound alterations in the bone marrow microenvironment, including chronic low-grade inflammation, immune dysregulation, and age-related cellular dysfunction (5, 6). In recent years, senescence has emerged as an important contributor to skeletal aging and osteoporosis. Senescent cells accumulate in bone tissues, secrete pro-inflammatory and tissue-remodeling mediators, and disrupt the coordinated activities of osteoblasts and osteoclasts, thereby accelerating bone loss (7–9). Thus, PMO should increasingly be understood as a disorder at the intersection of endocrine decline, immune remodeling, and senescence-associated tissue degeneration.

Among the immune cell populations involved in bone homeostasis, macrophages are particularly important. These cells are highly plastic and perform multiple roles in the skeletal microenvironment, including regulation of inflammatory tone, support of osteoblast differentiation, modulation of osteoclast genesis, and clearance of apoptotic cells during tissue remodeling (10, 11). Under physiological conditions, macrophages help maintain a balanced bone remodeling process. Under pathological conditions, however, macrophage dysfunction can shift the marrow niche toward persistent inflammation, defective tissue repair, and impaired osteogenic support. Emerging studies suggest that senescent macrophages are not merely bystanders in aging tissues, but active drivers of tissue degeneration because they display abnormal polarization, exaggerated inflammatory responses, and reduced efferocytosis capacity (12–14). In the skeleton, failure to effectively clear apoptotic osteoblasts and resolve local inflammatory stress may further compromise bone formation and aggravate osteoporosis (15–17). Nevertheless, the molecular regulators linking macrophage senescence to PMO progression remain insufficiently defined.

G protein-coupled receptor class C group 5 member B (GPRC5B) is an orphan G protein-coupled receptor that has attracted increasing attention because of its role in inflammatory regulation, immune-cell behavior, and disease-associated tissue remodeling (18). Previous studies have shown that GPRC5B is dynamically regulated in macrophages and may participate in inflammatory signaling and immune-cell recruitment (19, 20). In addition, evidence from skeletal and inflammatory disorders suggests that GPRC5B may influence pathological tissue responses under stress conditions (21). However, whether GPRC5B contributes to PMO, particularly through regulating macrophage dysfunction and senescence, has not been established. Clarifying this question may provide important insight into how immune remodeling is coupled to bone deterioration in PMO.

The downstream mechanism by which GPRC5B affects macrophage behavior is also unclear. DLG1 is a scaffold protein involved in organizing membrane-associated signaling complexes and regulating ion-channel stability, including that of the voltage-gated potassium channel Kv1.3 (22, 23). Kv1.3 has been implicated in inflammatory activation, immune-cell functional polarization, and senescence-associated signaling (24, 25). These observations raise the possibility that GPRC5B may regulate macrophage senescence through a DLG1/Kv1.3-dependent pathway.

In the present study, we integrated single-cell transcriptomics, bulk transcriptomic datasets, co-expression network analysis, machine learning, and experimental validation to identify macrophage-associated candidate regulators in PMO. Our findings implicate GPRC5B as a PMO-associated candidate enriched in disease-related macrophage populations and suggest that its effects may be linked to DLG1/Kv1.3-related signaling.

## Materials and methods

### Public datasets and bioinformatic analyses

Public datasets were downloaded from the Gene Expression Omnibus (GEO) database. Single-cell RNA-sequencing data from GSE147287 and GSE169396 were integrated for exploratory analysis of PMO-associated alterations. GSM4423510 from GSE147287 and GSM5201883 from GSE169396, both derived from older female patients with osteoporosis annotations, were assigned to the PMO-annotated group. The other four samples were included in the available comparator group. Because detailed menopausal status and uniform clinical diagnostic criteria were not available for all public samples, these group assignments were operational and the findings were interpreted as exploratory PMO-associated observations. Bulk transcriptomic datasets were analyzed in parallel. Because GPRC5B is expressed in the monocyte-macrophage lineage and circulating monocytes are precursors of tissue macrophages, these publicly available blood-based BMD cohorts were used as an accessible surrogate for testing GPRC5B. For GSE56815, which contains 80 samples from both pre- and postmenopausal women, only the postmenopausal subset (20 high-BMD and 20 low-BMD) was analyzed to match the postmenopausal focus of this study, and no samples were excluded for quality reasons; this subset was used for feature selection and diagnostic model construction. GSE7158 (14 high-PBM and 12 low-PBM) and GSE7429 (10 high-BMD and 10 low-BMD) were used in full for external validation. GSE7158 comprised 14 high-PBM (Peak bone mass) and 12 low-PBM samples from young adult women. GSE7429 comprised an external postmenopausal B-cell cohort, including 10 high-BMD and 10 low-BMD samples.

Single-cell data were processed using Seurat (v4.2.2). Cells with fewer than 200 detected genes, more than 5,000 detected genes, or mitochondrial gene content >10% were excluded. Data were normalized using *SCTransform*, and principal component analysis was performed for dimensionality reduction. After PCA and Harmony correction, *FindNeighbors*, *FindClusters*, and *RunUMAP* were performed using the first 15 Harmony-corrected dimensions for whole-cell clustering, with a final resolution of 0.5. Macrophage subclustering was performed using the first 10 Harmony-corrected dimensions, with a final resolution of 0.6. Candidate resolutions from 0.2 to 1.0 were evaluated using *clustree*. Potential doublets were identified and removed using *DoubletFinder*, and batch effects across datasets were corrected using Harmony. Cell types were annotated based on canonical marker genes in combination with *SingleR*. Differentially expressed genes for each cluster were identified using the *FindAllMarkers* function, and visualization was performed using ggplot2. Each macrophage subcluster was defined by its overall differentially expressed gene signature and was named after one representative marker that was relatively enriched in that subcluster. Bulk transcriptomic data were background-corrected and normalized using the *limma* package.

Cell-cell communication analysis was performed using *CellChat* (v1.5.0). A *CellChat* object was generated from the scRNA-seq data, and communication networks were inferred using the *aggregateNet* function. Signaling interactions were visualized using the *netVisual_circle* function, and pathway centrality was estimated using *netAnalysis_computeCentrality*. Single-cell metabolic activity was assessed using the *scMetabolism* package.

Given the established role of RAS/MAPK signaling in cellular senescence, KRAS signaling was selected as a candidate pathway. Its activity across cell populations was evaluated using the irGSEA framework, integrating eight scoring algorithms (AUCell, UCell, singscore, ssGSEA, JASMINE, VAM, scSE, and Viper). Pathway scores were calculated with the *irGSEA.score* function and integrated across cell populations using *irGSEA.integrate*. Cell-type significance was determined from the irGSEA integrated result, in which per-method Wilcoxon rank-sum tests (each cell type versus all others) were combined across the eight algorithms by robust rank aggregation (RRA). High-dimensional weighted gene co-expression network analysis (hdWGCNA) was performed on the single-cell dataset. The analysis was initialized with *SetupForWGCNA*, and metacells were constructed using *MetacellsByGroups* to reduce sparsity. A soft-thresholding power of 12 was selected to achieve scale-free topology, and genes were clustered into co-expression modules. Module eigengenes were calculated and correlated with macrophage subtypes to identify APOE-positive macrophage-associated modules.

To prioritize diagnostic genes, XGBoost and LASSO regression were performed using the GSE56815 cohort. XGBoost (xgboost package) was run with 100 boosting rounds (max_depth=4, eta=1), selecting the optimal iteration at the minimum log loss, and gene importance was ranked using xgb.importance. LASSO regression (glmnet package) used 10-fold cross-validation, with lambda selected at the minimum binomial deviance (lambda.min). Given the limited sizes of the cohorts, the machine-learning results serve to prioritize candidate genes. Genes with non-zero coefficients were retained. Shared genes identified by both algorithms were considered core diagnostic candidates. ROC curves were generated using the pROC package. Functional enrichment analysis of GPRC5B-associated genes was performed using clusterProfiler and visualized with GseaVis. Immune infiltration was analyzed by single-sample gene set enrichment analysis (ssGSEA) using the GSVA and GSEABase packages, and the enrichment scores of 29 immune cell types and immune-related functional signatures were quantified. Correlations between GPRC5B and chemokine/TNF family genes were visualized using ggplot2.

Potential compounds targeting GPRC5B were screened using the DSigDB database via the Enrichr platform. Compounds with adjusted P < 0.01 were considered significant, and the top 20 candidates were selected for further analysis. Molecular docking was performed to assess the interaction between the top-ranked compound and GPRC5B. The three-dimensional structure of GPRC5B was obtained from the Protein Data Bank. Protein and ligand structures were prepared using AutoDock Tools 1.5.6 by adding polar hydrogens, assigning Gasteiger charges, and defining rotatable bonds. A grid box covering the entire protein structure was applied, and docking simulations were carried out using AutoDock Vina 1.2.5. Docking conformations were visualized using PyMol (v3.2).

### Animal experiments

Female C57BL/6 mice were purchased from Changzhou Cavens Experimental Animal Co., Ltd. Female C57BL/6 mice were assigned to the age- and bilateral ovariectomy (OVX)-related experiments, with five mice in each group: young Sham, young OVX, aged Sham, and aged OVX. Two-month-old and 23-month-old mice underwent either bilateral ovariectomy or sham surgery and were euthanized four weeks after surgery, at approximately 3 and 24 months of age, respectively. Animals within each age stratum were allocated to the Sham or OVX group using simple random allocation. The procedure was performed as previously described (26). Briefly, mice were anesthetized by intraperitoneal injection of 3% sodium pentobarbital, fixed in a prone position, and subjected to bilateral removal of the ovaries, ovarian bursae, and part of the oviduct through a dorsal approach. Sham-operated mice underwent the same procedure without ovary removal. After surgery, mice were kept warm until recovery and received carprofen (5 mg/kg, intraperitoneal injection) for 3 consecutive days for analgesia.

For *in vivo* mechanistic studies, aged OVX mice were allocated to three groups, with five mice per group: PMO, PMO+AAV-shGPRC5B, and PMO+AAV-shGPRC5B+DLG1 inhibitor. OVX mice received intra-bone marrow injection of AAV-shGPRC5B (4.5×10^13^ vg/mL) one week after surgery to reduce GPRC5B expression within the bone marrow microenvironment (27). Two weeks after surgery, the DLG1 inhibitor AVLX-144 was administered by tail vein injection with 3 nmol/g dose based on previous *in vivo* studies (28, 29). At the experimental endpoint, all mice were euthanized by CO_2_ asphyxiation followed by cervical dislocation, and femurs were collected for micro-CT reconstruction and histological imaging. Investigators performing micro-CT reconstruction, histological imaging and image analysis were blinded to group allocation through sample coding.

### Micro-computed tomography

After euthanasia, femurs were harvested and fixed immediately in 4% paraformaldehyde. Samples were scanned using a Skyscan 1176 micro-CT system at 70 kVp and 114 μA with an isotropic voxel size of 10 μm. Raw micro-CT projection data were reconstructed using NRecon software. Reconstructed images were processed using CTAn software with a uniform grayscale threshold of 65–255 for bone segmentation across all samples. Three-dimensional reconstructed images were generated using CTVox software. The same threshold and reconstruction settings were applied to all groups.

### Immunofluorescence staining

Paraffin-embedded femoral sections were deparaffinized, rehydrated, and subjected to antigen retrieval in EDTA buffer. Sections were permeabilized with 0.5% Triton X-100 for 10 min and blocked with 3% bovine serum albumin for 1 h at room temperature. The sections were then incubated overnight at 4 °C with anti-F4/80 antibody (Abcam, ab6640) and anti-GPRC5B antibody (Novus Biologicals, NBP1-87160), followed by fluorescent secondary antibodies for 1 h at room temperature in the dark. Nuclei were counterstained with DAPI, and images were acquired using an OLYMPUS IX73 microscope.

### Isolation of primary bone marrow-derived macrophages

Primary bone marrow-derived macrophages (BMMs) were isolated from femurs and tibiae. Bone marrow cells were flushed with cold PBS, filtered, and collected by centrifugation. Red blood cells were removed using erythrocyte lysis buffer. To remove stromal cells, the suspension was incubated for 4 h to allow adherence. Non-adherent cells were then collected and cultured in RPMI 1640 medium supplemented with 30 ng/mL M-CSF for 7 days, with medium changes every 2 days, to induce macrophage differentiation.

### Cell culture and treatments

RAW264.7 cells were cultured in DMEM supplemented with 10% fetal bovine serum and 1% penicillin/streptomycin at 37 °C in a humidified atmosphere containing 5% CO2.

For gene silencing experiments, primary BMMs or RAW264.7 cells were transfected with siGPRC5B or negative control siRNA using Lipofectamine RNAiMAX for 24 h. To induce cellular senescence, RAW264.7 cells were treated with 100 μM H_2_O_2_ for 2 h following transfection (30). For rescue experiments, the full-length coding sequence of DLG1 was cloned into the pCDH-CMV-MCS-EF1-Puro lentiviral vector to generate pCDH-DLG1. RAW264.7 cells were co-transfected with siGPRC5B together with either pCDH-DLG1 or empty vector (oeNC) for 24 h, followed by treatment with 100 μM H_2_O_2_ for 2 h.

To mimic the estrogen-deficient microenvironment of PMO, RAW264.7 cells were cultured in phenol red-free DMEM (Procell, PM150278) supplemented with 10% charcoal-stripped fetal bovine serum (Sigma-Aldrich, F6765) and 1 μM estrogen receptor antagonist Fulvestrant/ICI 182,780 (MedChemExpress, HY-13636). Cells cultured in standard complete medium served as controls. To assess the pharmacological effect of Doramectin, estrogen-deprived RAW264.7 cells were treated with Doramectin (MedChemExpress, HY-17035; 10 mM stock solution in DMSO) at 0, 5, 10, or 20 μM for 24–48 h before RNA and protein analyses.

### Western blot

Total protein was extracted from tissues or cells using RIPA lysis buffer, quantified using the BCA assay, and denatured by boiling. Equal amounts of protein were separated by SDS-PAGE and transferred onto PVDF membranes. After blocking, membranes were incubated overnight at 4 °C with primary antibodies against GPRC5B (Thermo Fisher, MA5-63445), DLG1 (BD Biosciences, 610874), Kv1.3 (Alomone Labs, APC-101-B), and β-actin (Abcam, ab8226). Membranes were then incubated with the corresponding secondary antibodies for 1 h at room temperature. Protein signals were detected using enhanced chemiluminescence and imaged with a chemiluminescence imaging system.

### Senescence-associated β-galactosidase staining

Cells were washed with PBS, fixed with β-Gal staining fixative for 15 min at room temperature, and washed three times with PBS. β-galactosidase staining was performed using a Cellular Senescence β-Galactosidase Staining Kit (C0602, Beyotime). Freshly prepared β-galactosidase staining working solution was then added according to the manufacturer’s instructions, and cells were incubated overnight at 37 °C in a dry incubator without CO_2_. For quantification of SA-β-gal staining, images were analyzed using ImageJ. SA-β-gal-positive cells were defined as blue-stained cells, and the percentage of senescent cells was calculated as: SA-β-gal-positive cells/total cells×100%.

### Flow cytometry

Macrophage polarization was assessed by flow cytometry. Single-cell suspensions were incubated with anti-CD86 antibody (BioLegend, 159202), anti-CD206 antibody (BioLegend, 141713), or corresponding isotype controls at 4 °C for 30 min in the dark. After washing, cells were resuspended in staining buffer and analyzed on a flow cytometer (Beckman Coulter Life Sciences). Data were processed using FlowJo software, and the proportions of CD86-positive (M1) and CD206-positive (M2) macrophages were quantified.

### RT-qPCR

Total RNA was extracted using a commercial RNA isolation kit, and 1 μg RNA was reverse-transcribed into cDNA. Quantitative PCR was performed using SYBR Premix Ex Taq on a real-time PCR system. Relative mRNA expression was calculated using the 2^-ΔΔCt^ method with GAPDH as internal control. The primers were as follows: GPRC5B forward: 5’-AGTGCACCGTTCAGAAGCAA-3’, reverse: 5’-GGACAGCCATTTCAGTCCCT-3’; IL-1β forward: 5’-TGGCAACTGTTCCTG-3’, reverse: 5’-GGAAGCAGCCCTTCATCTTT-3’; GAPDH forward: 5’-AAGAGGGATGCTGCCCTTAC-3’; reverse: 5’-TACGGCCAAATCCGTTCACA-3’.

### ELISA

Culture supernatants from RAW264.7 cells under control or estrogen-deprived conditions were collected, and the concentrations of TNF-α and IL-10 were measured using commercial ELISA kits according to the manufacturers’ instructions. TNF-α levels were determined using a mouse TNF-α ELISA kit (Reddot Biotech, RE1060MF), and IL-10 levels were measured using a mouse IL-10 ELISA kit (Multisciences, EK210).

### Phagocytosis assay

To assess macrophage phagocytosis of apoptotic osteoblasts, MC3T3-E1 cells were treated with 1 μM dexamethasone for 24 h to induce apoptosis (31) and labeled with Sytox Green (Invitrogen, S7020). RAW264.7 cells were pre-labeled with CellTracker Red CMTPX (Invitrogen, C34552). Labeled apoptotic MC3T3-E1 cells and CellTracker Red-labeled RAW264.7 macrophages were co-cultured at a 1:1 ratio for 3 h. After extensive washing to remove non-engulfed cells, the samples were fixed, counterstained with DAPI, and imaged using identical microscope settings. The assay was performed in three independent biological replicates. For each replicate, five randomly selected non-overlapping fields were analyzed. A phagocytosis-positive macrophage was defined as a CellTracker Red-positive cell containing at least one Sytox Green-positive apoptotic osteoblast signal clearly enclosed within the macrophage boundary, producing an intracellular yellow merged signal. Externally adherent green signals without clear evidence of internalization were excluded. The percentage of phagocytosis-positive macrophages was calculated as follows: phagocytosis-positive macrophages/total CellTracker Red-positive macrophages×100%.

### Co-immunoprecipitation

Co-immunoprecipitation (Co-IP) was performed to assess the interaction between GPRC5B and DLG1. Briefly, 293T cells were lysed with RIPA buffer, and cell lysates were incubated overnight at 4 °C with anti-GPRC5B antibody. Protein A agarose beads were then added to capture immune complexes. After washing, complexes were eluted by boiling in denaturing buffer and analyzed by western blot.

### Statistical analysis

All data are presented as mean ± standard deviation (SD). Statistical analyses were performed using GraphPad Prism. Comparisons between two groups were conducted using Student’s t-test. Comparisons among multiple groups were analyzed by one-way or two-way analysis of variance (ANOVA) followed by Tukey’s *post hoc* test. All experiments were independently repeated at least three times. A value of P < 0.05 was considered statistically significant.

## Results

### Single-cell transcriptomic profiling reveals APOE-positive macrophages as a PMO-enriched subset with elevated KRAS-related signaling

**Figure 1:**
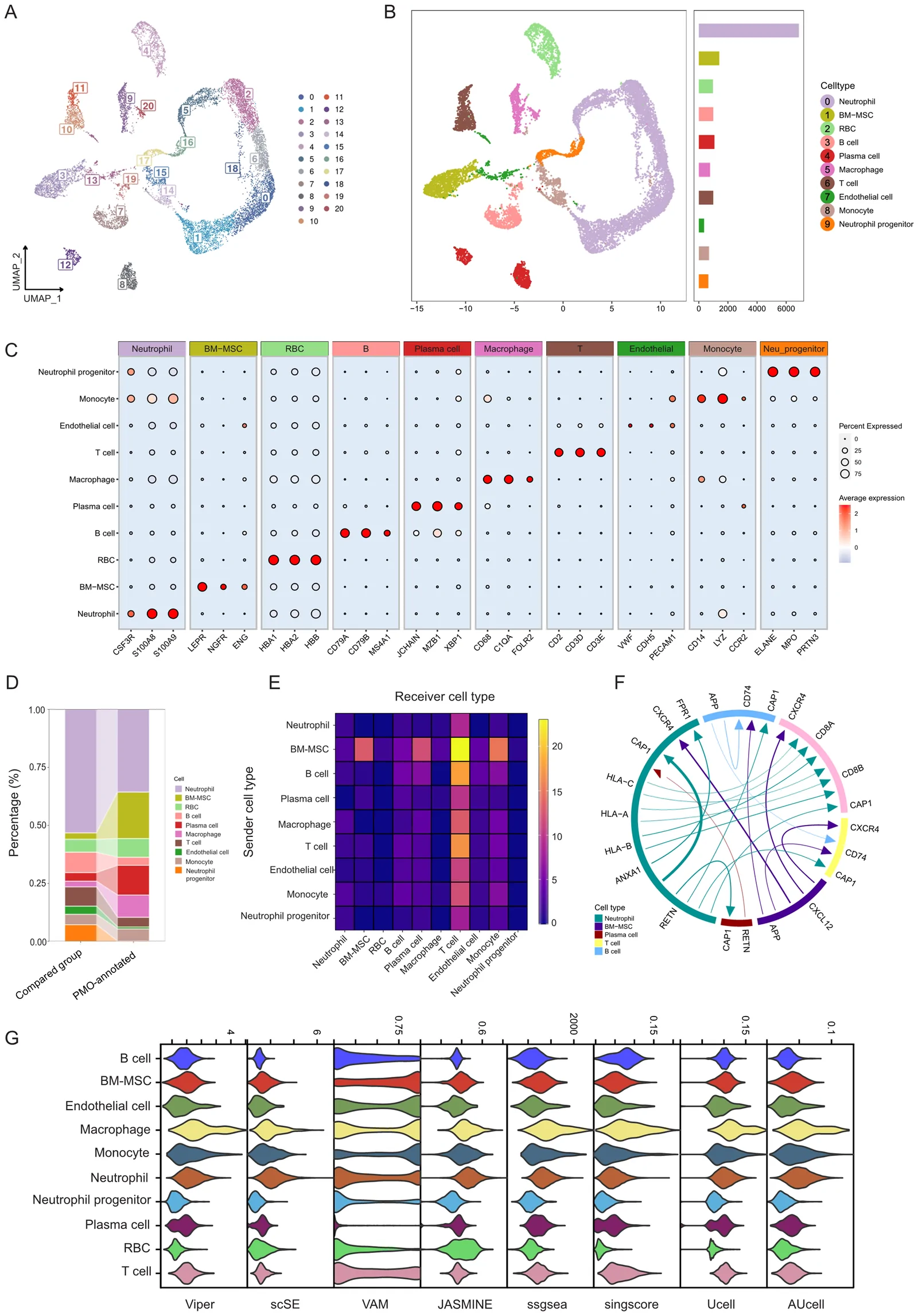
Single-cell landscape of post-menopausal osteoporosis. **(A)** UMAP plot showing 14,809 cells profiled by single-cell RNA-sequencing. Following initial quality control, cells were partitioned into 21 clusters (0-20). Each dot represents a cell, and clusters are color-coded. **(B)** UMAP plot with 10 cell types (0-9) colored according to their high expression genes. Cell types include neutrophils, BM-MSC (bone marrow-derived mesenchymal stem cells), RBC (red blood cells), B cells, plasma cells, macrophages, T cells, endothelial cells, monocytes and Neutrophil progenitor. **(C)** Dot plot showing the expression of marker genes across 10 different cell types. The size of the dot represents the percentage of cells expressing the gene, and the color represents the average expression level. **(D)** Proportion of each cell type in available compared samples and PMO-annotated samples based on public dataset annotations. Stacked bar plots show the percentage of each cell type in the two groups. Sample groups were defined based on the original public dataset annotations. The available comparator samples were not healthy controls and included individuals with heterogeneous clinical backgrounds. **(E)** Cell communication network. The network plot shows the signaling interactions between different cell types. The color indicates the number of interactions. **(F)** Circular plot showing cell-cell communication centered on specific signaling molecules. The outer rings represent cell types, and the inner lines show the signaling pathways between them. **(G)** KRAS signaling enrichment across cell types. The eight panels show the raw per-algorithm score distributions; cell-type ranking and significance were determined from the irGSEA RRA-integrated result across all eight algorithms, in which macrophages ranked first.

Single-cell RNA sequencing of bone marrow samples from PMO-annotated samples and available compared samples identified 14,809 high-quality cells, which were classified into 21 clusters and further annotated into 10 major cell types (**Figures 1A–C**). Compared with the available samples, PMO-annotated samples showed apparent remodeling of cellular composition, with increased BM-MSCs, plasma cells, and macrophages, together with reduced neutrophils, B cells, and neutrophil progenitors (**Figure 1D**). Cell-cell communication analysis further revealed extensive niche rewiring in PMO, with BM-MSCs acting as a major signaling source and T cells as major signaling recipients; ANXA1-CAP1 was among the prominent ligand-receptor pairs (**Figures 1E, F**). Consistent with the known involvement of RAS/MAPK signaling in senescence, KRAS signaling activity was highest in macrophages among other cell populations (**Figure 1G**), supporting a macrophage-focused analysis.

**Figure 2:**
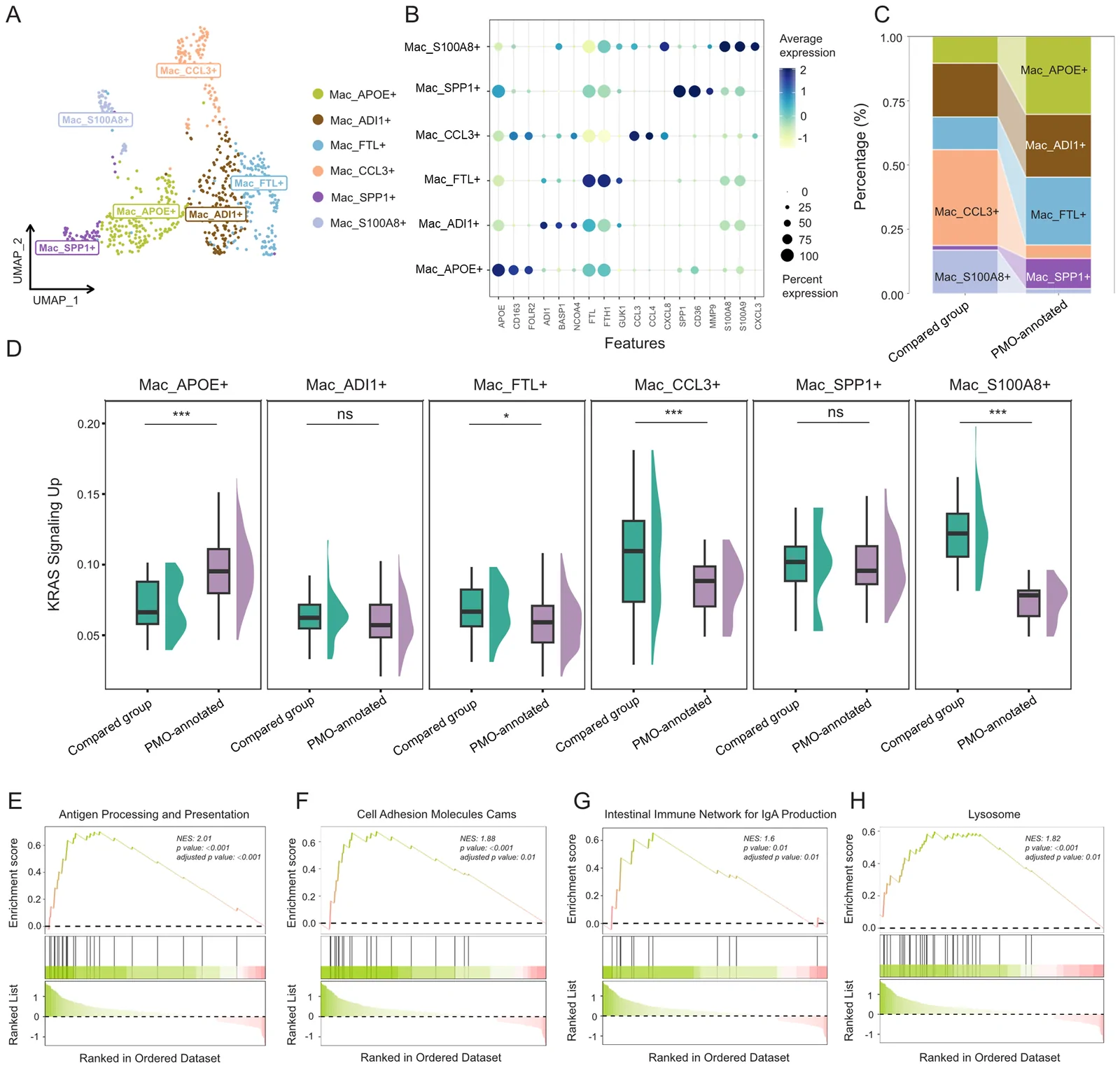
Macrophage subclustering and KRAS pathway activation. **(A)** UMAP plot of macrophage subtypes. Each dot represents a cell, and color indicates the subtype. Six subclusters were identified: APOE+, ADI1+, FTL+, CCL3+, SPP1+ and S100A8 +. **(B)** Dot plot showing the expression of marker genes in macrophage subtypes. The size of the dot represents the percentage of cells expressing the gene, and the color represents the average expression level. **(C)** Proportion of macrophage subtypes in PMO-annotated and available compared group. Stacked bar plots show the percentage of each subtype in the two groups. **(D)** KRAS pathway activation in six macrophage subtypes. The plot shows the KRAS pathway activation score in each subtype for PMO-annotated and available compared groups. Significant differences are indicated by asterisks. **(E–H)** GOBP enrichment analysis of APOE+ macrophages. The plots show the enriched biological processes in APOE+ macrophages, including antigen processing and presentation **(E)**, cell adhesion molecules **(F)**, intestinal immune network for IgA production **(G)**, and lysosome **(H)**. *p < 0.05, ***p < 0.001; ns, not significant.

Re-clustering of macrophages identified six subpopulations, among which APOE+, FTL+ and SPP1+ macrophages were enriched in PMO (**Figures 2A–C**). APOE+ macrophages showed significantly higher KRAS pathway activity in the PMO-annotated group than in the compared group (**Figure 2D**) and were enriched in antigen presentation, cell adhesion, IgA-related immune responses, and lysosomal pathways (**Figures 2E–H**). To further define the molecular features of this subset, hdWGCNA identified a gene module most strongly associated with APOE+ macrophages, and intersection with KRAS pathway genes yielded six candidate genes: ANKH, GYPC, GPRC5B, LY96, FUCA1, and TMEM176B (**Supplementary Figure 1**).

### Integrated transcriptomic and machine-learning analyses prioritize GPRC5B as a macrophage-enriched PMO-associated candidate

**Figure 3:**
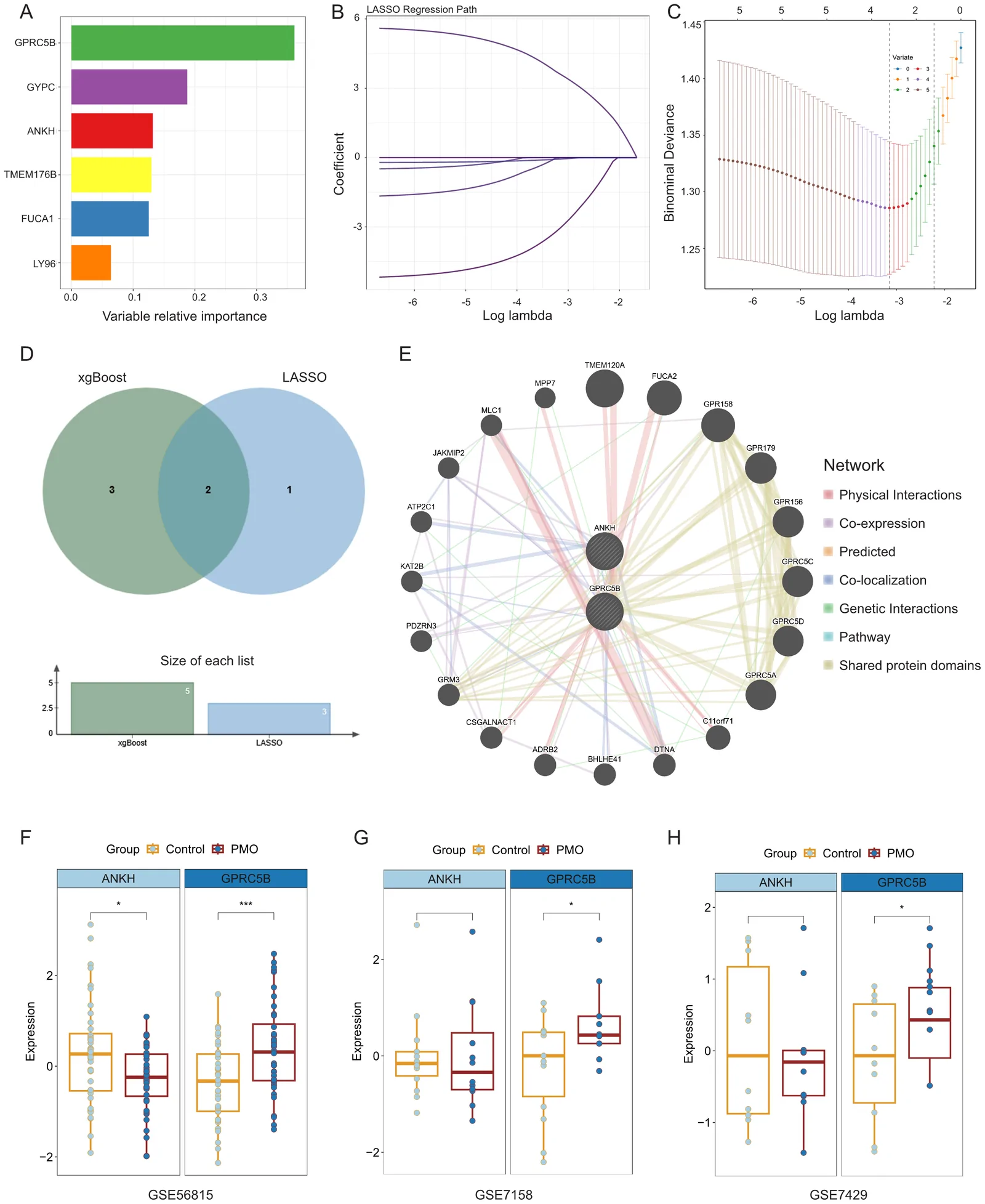
Identification and validation of diagnostic genes. **(A)** Bar plot of variable relative importance from XGBoost, displaying the relative importance of different variables with the height of each bar representing the degree of importance. **(B)** LASSO regression path plot illustrates the coefficient profiles of variables as the regularization parameter (Log lambda) changes in LASSO regression. When coefficients shrink, some variables are eliminated as lambda increases. **(C)** Binomial deviance plot for LASSO regression. It shows the relationship between the log of the lambda parameter and the binomial deviance, helping to determine the optimal lambda that balances model fit and complexity. **(D)** Venn diagram showing the intersection of XGBoost and LASSO results. The Venn diagram shows the overlap between genes selected by XGBoost and LASSO regression. **(E)** Protein-protein interaction network of core genes. The network plot shows the interactions between the core diagnostic genes. **(F–H)** Box plots showing gene expression across GSE56815 **(F)**, GSE7158 **(G)**, and GSE7429 **(H)** datasets. The box plots show the expression levels of ANKH and GPRC5B in PMO and control group. *p < 0.05, ***p < 0.001.

To prioritize key genes, we integrated XGBoost and LASSO analyses, which identified ANKH and GPRC5B as shared candidate genes, with GPRC5B ranked highest by XGBoost (**Figures 3A–D**). The protein-protein interaction network depicts the interactions between these core genes, suggesting their potential coordinated role in regulating PMO-associated macrophage functions (**Figure 3E**). In three independent PMO datasets, GPRC5B was consistently upregulated, whereas ANKH showed less stable expression changes (**Figures 3F–H**). Receiver operating characteristic analysis showed consistent discriminatory performance across datasets (GSE56815, AUC = 0.812, 95% CI 0.677-0.947; GSE7158, AUC = 0.780, 95% CI 0.601-0.959; GSE7429, AUC = 0.700, 95% CI 0.466-0.934; **Supplementary Figures 2A–C**), supporting GPRC5B as a candidate diagnostic marker for PMO.

Further analyses showed that GPRC5B was closely associated with immune-related pathways and immune infiltration features, including macrophages, T-cell signatures, MHC molecules, inflammatory responses, chemokines, and TNF family members (**Supplementary Figures 2D–F**; **Supplementary Figure 3**). Although GPRC5B is not a marker that defines the APOE+ cluster, single-cell mapping showed that its expression was relatively higher in macrophages and comparatively highest in the APOE+ subset, consistent with its identification through co-expression and machine-learning analysis (**Supplementary Figures 4A–D**). In addition, GPRC5B-positive macrophages displayed enhanced intercellular communication with multiple cell populations, particularly neutrophils and T cells (**Supplementary Figures 4E, F**), suggesting that GPRC5B marks a distinct macrophage state in PMO.

### GPRC5B is upregulated in PMO-associated macrophages and is linked to senescence-associated and functional changes

**Figure 4:**
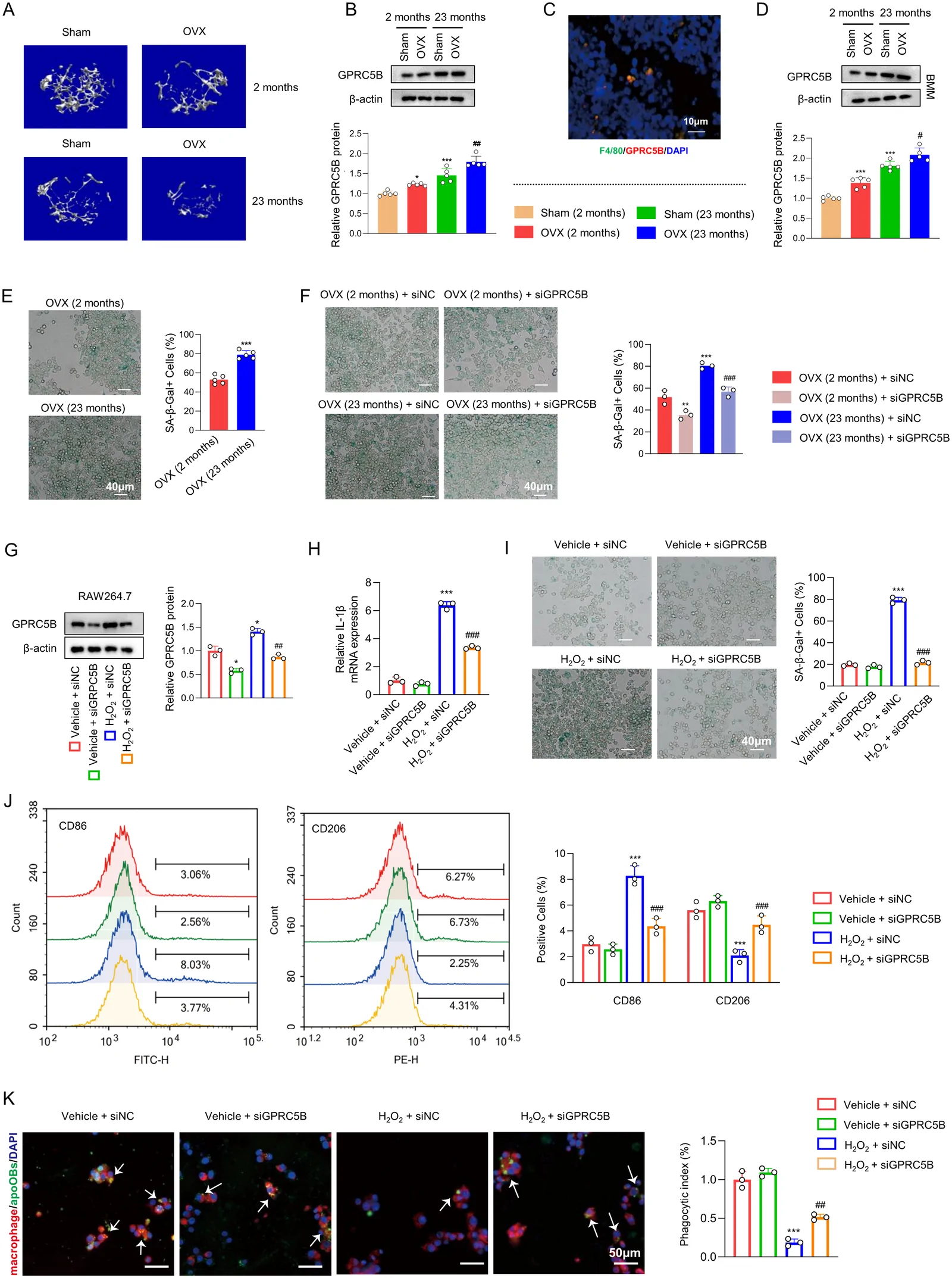
GPRC5B upregulation is associated with macrophage senescence-related changes in a PMO mouse model. A post-menopausal osteoporosis (PMO) mouse model was established by ovariectomy (OVX). 2-month-old and 23-month-old mice were randomly assigned to different groups (n=5): the experimental group (OVX) and the sham-operated (Sham) group. GPRC5B expression and its effects on macrophage senescence were subsequently evaluated in bone marrow-derived macrophages (BMMs) and RAW264.7 cells. **(A)** Representative three-dimensional reconstructed micro-CT images of femurs from one mouse of different group. **(B)** Western blot analysis of GPRC5B protein expression in bone marrow tissue. **(C)** Immunofluorescence detection of GPRC5B expression and localization in femoral tissue macrophages (F4/80+). This staining was used to confirm GPRC5B is localized in macrophages (F4/80+), which is not restricted to a single macrophage subset or being used to assess overall tissue architecture. **(D)** Western blot analysis of GPRC5B protein expression in primary BMMs. **(E)** β-Galactosidase (β-Gal) staining of primary BMMs isolated from femurs of 2- and 23-month OVX mice to assess cellular senescence (n=5). **(F)** β-Gal staining of BMMs following transfection with siGPRC5B to evaluate the effect of GPRC5B knockdown on senescence (n=3). For panels B-F, statistical symbols placed above an individual group indicate comparison of that group with the specified reference group: ^***^p < 0.001 versus the 2-month-old Sham group; ^#^p < 0.05 and ^###^p < 0.001 versus the 23-month-old Sham group. **(G–K)** RAW264.7 cells were transfected with siGPRC5B and subsequently treated with H_2_O_2_ to induce senescence. Cells were divided into four groups (n=3): Vehicle+siNC, Vehicle+siGPRC5B, H_2_O_2_+siNC, and H_2_O_2_+siGPRC5B. **(G)** Western blot analysis of GPRC5B protein expression in RAW264.7 cells after siGPRC5B transfection. **(H)** RT-qPCR analysis of IL-1β mRNA expression levels. **(I)** β-Gal staining to assess cellular senescence. **(J)** Flow cytometry analysis of macrophage polarization, showing CD86-positive (M1) and CD206-positive (M2) populations. **(K)** Representative fluorescence images showing phagocytosis of apoptotic osteoblasts by macrophages. Yellow merged signals indicate apoptotic osteoblast uptake by macrophages. White arrows indicate selected representative engulfment events and do not represent all phagocytic macrophages in the field. For panels G-K, statistical symbols placed above an individual group indicate comparison of that group with the specified reference group: ^***^p < 0.001 versus the Vehicle+siNC group; ^###^p < 0.001 versus the H_2_O_2_+siNC group.

Given the enrichment of GPRC5B in macrophages, we next investigated its relevance to estrogen deficiency and aging-associated PMO. An ovariectomy (OVX)-induced PMO model was established in young (2-month-old) and aged (23-month-old) mice. Representative micro-CT reconstructions showed bone structural deterioration after OVX and aging, with the apparent trabecular bone loss observed in aged OVX mice (**Figure 4A**). Compared with the 2-month-old sham group, GPRC5B was significantly upregulated in both the 2-month-old OVX group and the 23-month-old sham group; compared with the 23-month-old sham group, GPRC5B expression was also significantly increased in the 23-month-old OVX group (**Figure 4B**), and immunofluorescence further confirmed GPRC5B expression in F4/80+ macrophages within femoral tissue (**Figure 4C**). Similar results were obtained in primary BMMs isolated from these mice (**Figure 4D**). These findings indicate that GPRC5B expression is significantly upregulated in macrophages under PMO conditions, influenced by aging and estrogen deficiency.

We next examined whether GPRC5B contributes to macrophage senescence. SA-β-gal staining showed increased senescence-associated β-galactosidase activity in primary BMMs from aged OVX mice, which was reduced following GPRC5B knockdown (**Figures 4E, F**). In RAW264.7 cells, H_2_O_2_ treatment induced cellular senescence, whereas GPRC5B silencing effectively reduced GPRC5B expression (**Figure 4G**), suppressed IL-1β expression (**Figure 4H**), decreased β-Gal-positive cells (**Figure 4I**), and attenuated the shift toward CD86-Positive M1 polarization while partially restoring CD206-Positive M2 features (**Figure 4J**). Moreover, fluorescence tracing revealed that GPRC5B knockdown altered the interaction of senescent macrophages with apoptotic osteoblasts, as evidenced by enhanced phagocytic uptake (**Figure 4K**). These data suggest that GPRC5B is associated with senescence-related, inflammatory and functional changes in macrophages under PMO-related stress conditions.

### GPRC5B-associated macrophage phenotypes involve DLG1/Kv1.3-related signaling

**Figure 5:**
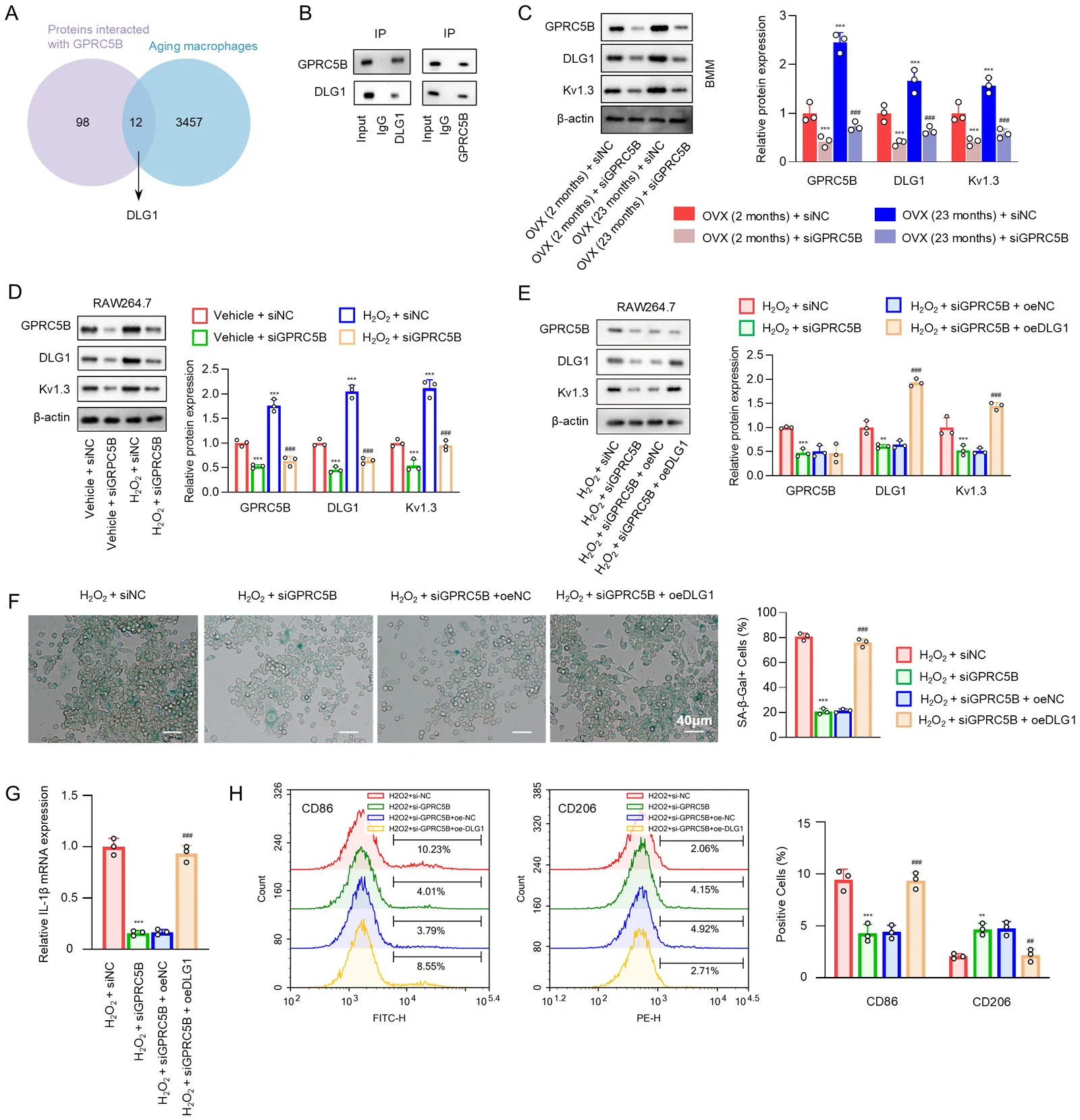
GPRC5B-associated macrophage changes involve DLG1/Kv1.3-related signaling. **(A)** Intersection analysis of GPRC5B-interacting proteins and targets related to senescent macrophages. **(B)** Co-IP validation of the interaction between GPRC5B and DLG1. **(C, D)** Western blot analysis of GPRC5B, DLG1, and Kv1.3 protein expression in BMMs from PMO mice and in RAW264.7 cells after GPRC5B knockdown. **(E–H)** RAW264.7 cells were divided into four groups (n=3): H_2_O_2_+siNC, H_2_O_2_+siGPRC5B, H_2_O_2_+siGPRC5B+oeNC, H_2_O_2_+siGPRC5B+oeDLG1. **(E)** Western blot analysis of GPRC5B, DLG1, and Kv1.3 protein expression levels in cells. **(F)** β-Gal staining of RAW264.7 cells. **(G)** RT-qPCR analysis of IL-1β mRNA expression levels. **(H)** Flow cytometry analysis of the proportions of CD86-Positive (M1) and CD206-Positive (M2) macrophages in different groups. ^**^p < 0.01, ^***^p < 0.001 vs. H_2_O_2_+siNC; ^##^*p* < 0.01, ^###^*p* < 0.001 vs. H_2_O_2_+siGPRC5B+oeNC.

To investigate the mechanism underlying GPRC5B-mediated macrophage senescence, we intersected GPRC5B-interacting proteins predicted by HitPredict with macrophage senescence-related targets from GeneCards, yielding 12 candidate proteins (**Figure 5A**, Datasets and genes in **Supplementary Tables**). Among these candidates, co-immunoprecipitation supported an association between GPRC5B and DLG1 within the same protein complex (**Figure 5B**). Western blot analysis showed that GPRC5B knockdown reduced the expression of DLG1 and its downstream channel protein Kv1.3 in both primary BMMs from both young and aged PMO mice (**Figure 5C**), suggesting an association between GPRC5B expression and DLG1/Kv1.3-related signaling.

To further examine the potential functional involvement of DLG1/Kv1.3-related signaling, we performed rescue experiments in H_2_O_2_-treated RAW264.7 cells. GPRC5B silencing reduced DLG1 and Kv1.3 expression (**Figure 5D**), while overexpression DLG1 increased DLG1 and Kv1.3 level (**Figure 5E**). GPRC5B knockdown attenuated β-Gal staining (**Figure 5F**), suppressed IL-1β expression (**Figure 5G**), and inhibited M1 polarization while promoting M2 features (**Figure 5H**). Importantly, DLG1 overexpression largely reversed these effects. These results indicate that GPRC5B-associated macrophage senescence-related changes may involve DLG1/Kv1.3-related signaling, where its knockdown impairs senescence-related phenotypes, and DLG1 overexpression reverses these effects.

### GPRC5B knockdown improves bone phenotypes, and Doramectin is identified as a candidate compound associated with GPRC5B expression

**Figure 6:**
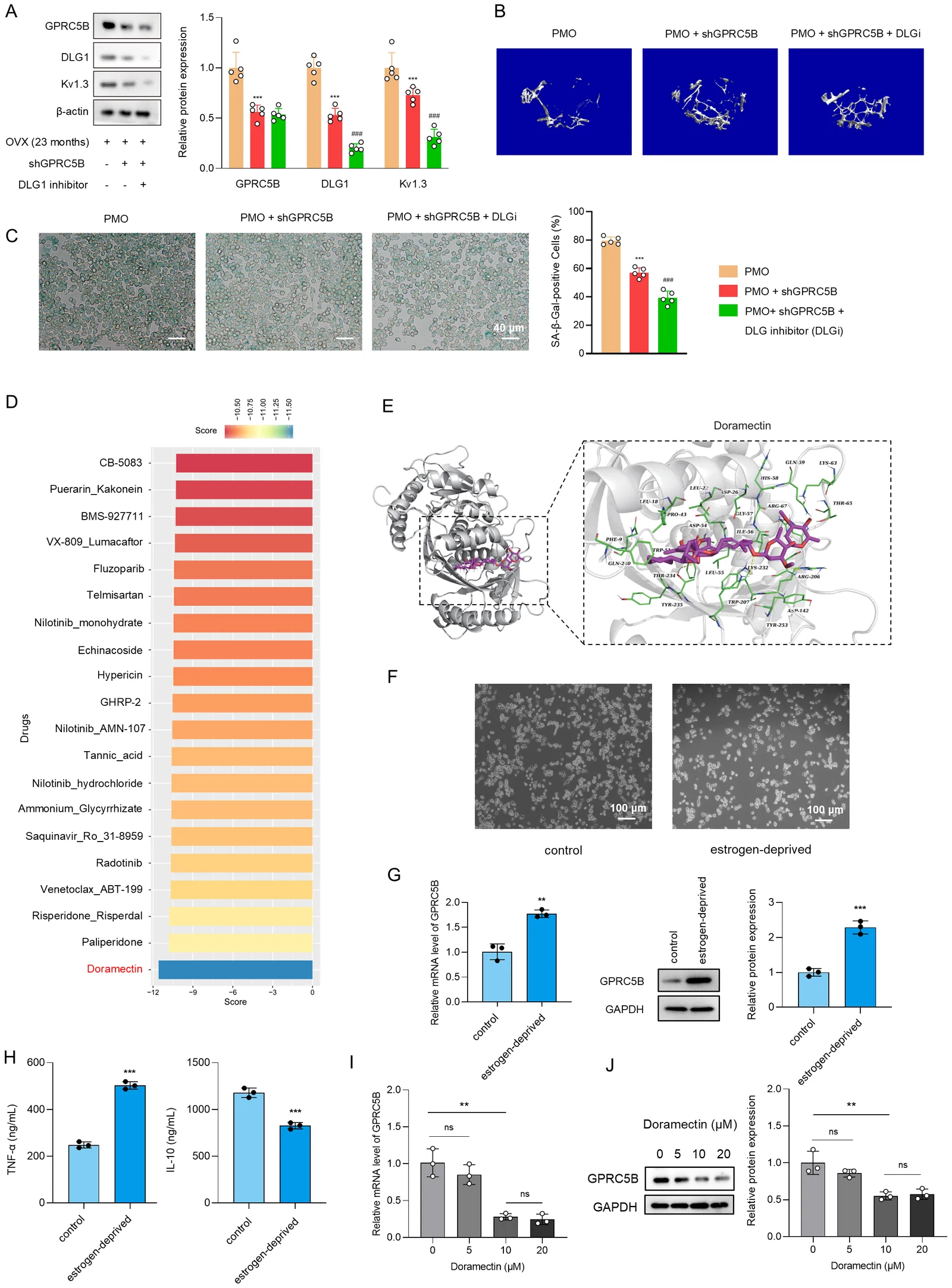
GPRC5B knockdown ameliorates PMO-associated phenotypes and reveals potential drug targets. GPRC5B-knockdown PMO mice were established and divided into three groups (n=5): PMO (23 months OVX model), PMO+shGPRC5B, and PMO+shGPRC5B+DLG1 inhibitor (DLG1i). The effects of GPRC5B knockdown on bone phenotype, macrophage senescence, and potential drug interactions were subsequently evaluated. **(A)** Western blot analysis of GPRC5B, DLG1, and Kv1.3 protein expression levels in primary bone marrow-derived macrophages from each group. **(B)** Representative three-dimensional reconstructed micro-CT images of mice femurs. **(C)** β-Gal staining of primary BMMs to assess cellular senescence. ***p < 0.001 vs. PMO; ###p < 0.001 vs. PMO+shGPRC5B. **(D)** Bar plot showing the scores of the top 20 drugs predicted to target GPRC5B using DSigDB. **(E)** Molecular docking of the top predicted drug, Doramectin, with GPRC5B, showing the 3D binding conformation. RAW264.7 macrophage cells were cultured and divided into two groups: control (normal medium) and estrogen deprivation. **(F)** Representative images of RAW264.7 cells under control and estrogen-deprived conditions. **(G)** RT-qPCR analysis of GPRC5B mRNA levels and western blot analysis of protein expression in RAW264.7 cells under control and estrogen-deprived groups. **(H)** ELISA detection of secreted inflammatory cytokines in culture supernatants, including pro-inflammatory TNF-α and anti-inflammatory IL-10. **(I, J)** Effects of Doramectin treatment (0, 5, 10, 20 μM for 24–48 h) on GPRC5B expression in RAW264.7 cells, assessed by RT-qPCR **(I)** and Western blot **(J)**.

To determine the *in vivo* role of GPRC5B, we established GPRC5B-knockdown PMO mice. Western blot of primary BMMs confirmed that GPRC5B knockdown reduced GPRC5B, DLG1 and Kv1.3 protein expression, and this effect was further enhanced by DLG1 inhibition (**Figure 6A**). Representative micro-CT reconstructions indicated a trend toward improved bone structural preservation after AAV-mediated GPRC5B knockdown, with a further apparent improvement in the DLG1 inhibitor co-treatment group. (**Figure 6B**). Consistently, SA-β-gal staining showed reduced senescence-associated β-galactosidase activity in primary BMMs after GPRC5B knockdown, with a further reduction upon co-treatment with the DLG1 inhibitor (**Figure 6C**). These findings show that intra-bone marrow GPRC5B knockdown was accompanied by reduced macrophage senescence-related signals, lower DLG1 and Kv1.3 expression, and a trend toward improved bone structural preservation.

To identify potential compounds targeting GPRC5B, we next performed drug prediction using DSigDB. Among the top 20 candidate compounds from the unbiased DSigDB screen, Doramectin achieved the highest prediction score (**Figure 6D**) and was applied to explore whether GPRC5B can be targeted. Molecular docking further supported stable binding of Doramectin to GPRC5B (**Figure 6E**). To mimic the endocrine component of PMO, RAW264.7 cells were cultured under estrogen-deprived conditions. Under these conditions, macrophages exhibited subtle morphological alterations, including a more rounded appearance, reduced spreading, and fewer visible cellular protrusions compared with control cells (**Figure 6F**), increased GPRC5B mRNA and protein expression (**Figure 6G**), and changes in cytokine secretion, including increased TNF-α and reduced IL-10 level (**Figure 6H**). Importantly, Doramectin treatment suppressed GPRC5B expression in a dose-dependent manner, with significant inhibition observed at 10 μM and no further obvious reduction at 20 μM (**Figures 6I, J**). These findings are exploratory and nominate Doramectin as a compound of interest for future target-engagement and functional studies.

## Discussion

In the present study, we combined transcriptomic profiling, computational screening, and experimental validation to define a macrophage-centered mechanism underlying PMO progression. Our results show that PMO is accompanied by marked remodeling of the bone marrow microenvironment and substantial changes in macrophage state. Among the molecules identified through integrated multi-omics analysis, GPRC5B emerged as a potential candidate because it was consistently upregulated across datasets, enriched in disease-associated macrophage populations, and functionally linked to macrophage senescence and bone loss. Mechanistically, we found that GPRC5B was associated with DLG1/Kv1.3-related signaling and with senescence-related macrophage changes. In PMO mice, intra-bone marrow GPRC5B knockdown was accompanied by an apparent trend toward improved bone structure and reduced macrophage senescence, an effect further enhanced by DLG1 inhibition. Together, these findings are consistent with a model in which GPRC5B may link marrow immune remodeling to skeletal deterioration in PMO.

One important implication of this study is that PMO should not be understood solely as a disorder of estrogen withdrawal and osteoclast overactivation. Rather, our findings reinforce the concept that PMO involves a broader disruption of the marrow immune microenvironment. Increasing evidence indicates that low-grade chronic inflammation and altered immune-cell function contribute substantially to age-related bone loss (5, 6). The bone marrow is not merely a passive structural reservoir, but a highly dynamic niche in which stromal cells, immune cells, and bone cells continuously interact. Once this microenvironment becomes chronically dysregulated, signals that normally support tissue repair and balanced remodeling may instead favor inflammatory persistence, senescence accumulation, and impaired osteogenesis. Our single-cell analysis is consistent with this framework, revealing broad cellular compositional changes and communication rewiring in PMO-annotated samples derived from public datasets. These observations provide additional support for the growing osteoimmunology view that immune remodeling is not secondary to osteoporosis, but integral to its pathogenesis. However, it should be noted that these observations are based on publicly available datasets with heterogeneous clinical backgrounds and without ideal healthy controls, and therefore should be interpreted as exploratory PMO-associated findings.

Among marrow immune populations, macrophages are uniquely positioned to influence bone remodeling because they integrate inflammatory, phagocytic, and trophic functions (10, 32–34). In physiological bone, macrophages help sustain osteoblast activity, resolve local stress responses, and remove apoptotic debris. Under aging- and PMO-related stress, however, macrophages may shift into dysfunctional states characterized by persistent inflammatory activation, defective clearance capacity, and impaired support for tissue regeneration. Our experimental results strongly support this idea. In both primary macrophages and RAW264.7 cells, suppression of GPRC5B attenuated senescence-associated β-galactosidase activity, reduced IL-1β expression, improved clearance of apoptotic osteoblasts, and shifted macrophages away from a CD86-positive M1-like phenotype. These findings suggest that macrophage senescence in PMO is not simply reflected by growth arrest markers, but is accompanied by a broader program of inflammatory and functional imbalance. This broader view is important because it links macrophage senescence directly to impaired bone homeostasis.

A key contribution of our study is the use of single-cell analysis to dissect disease-associated macrophage heterogeneity in PMO. Rather than treating macrophages as a uniform population, our data indicate that PMO is associated with the emergence or expansion of transcriptionally distinct macrophage states with heightened inflammatory and signaling activity. Among these, one disease-associated population displayed prominent KRAS pathway activation and immune-remodeling features. Although the specific subcluster identity was informative for discovery, the broader biological message is more important: PMO selectively enriches macrophage states that are coupled to pathological signaling programs rather than simply increasing macrophage number. This interpretation is likely to be more relevant biologically than focusing narrowly on a single marker-defined subset. KRAS signaling, while classically studied in cancer biology, is increasingly recognized as a regulator of immune-cell activation, metabolic adaptation, and stress responses in non-malignant tissues (35, 36). In our dataset, enhanced KRAS signaling in disease-associated macrophages was accompanied by enrichment of antigen presentation, lysosomal, and adhesion-related pathways, indicating that these cells may act as active hubs of immune remodeling in the osteoporotic marrow environment.

Within this disease-associated macrophage landscape, GPRC5B stood out as the most relevant molecular candidate. It was identified by integrating co-expression network analysis with machine-learning approaches and subsequently showed reproducible upregulation in multiple validation cohorts. This is an important strength of the study because many candidate biomarkers emerging from transcriptomic analyses fail to reproduce across independent datasets. By contrast, GPRC5B showed both stability and functional relevance. Existing literature has suggested that GPRC5B participates in macrophage biology and inflammatory regulation (18–20), but its role in osteoporosis has remained largely undefined. Our study extends this knowledge by suggesting that GPRC5B is not merely a disease-associated marker, but may functionally contribute to macrophage dysfunction under PMO-related stress conditions. This distinction is critical. Biomarkers may be clinically useful, but molecules that are both diagnostically informative and mechanistically involved offer much greater translational value.

Another notable observation was that GPRC5B upregulation under estrogen-deprived conditions was accompanied by altered cytokine secretion. Specifically, estrogen-deprived macrophages exhibited increased TNF-α and reduced IL-10 levels, indicating a shift toward a more pro-inflammatory immune state. This finding was directionally consistent with the increased IL-1β expression and enhanced CD86-positive M1-like features observed in the H_2_O_2_-induced stress model. The estrogen deprivation model as well as oxidative stress model together suggest that elevated GPRC5B expression is associated with inflammatory and dysfunctional macrophage phenotypes. Nevertheless, further studies using additional cytokines and functional readouts are required to define the relationship between GPRC5B and macrophage inflammatory regulation.

Mechanistically, our data suggest that the DLG1/Kv1.3 axis is involved in the macrophage phenotypes associated with GPRC5B under PMO-related stress conditions. DLG1 is known to scaffold membrane-associated signaling complexes and regulate ion-channel localization and stability. Kv1.3, in turn, has been implicated in immune-cell activation, redox balance, and senescence-related signaling. We found that GPRC5B physically interacted with DLG1 and that GPRC5B silencing reduced the expression of both DLG1 and Kv1.3. More importantly, DLG1 overexpression reversed the beneficial effects of GPRC5B knockdown on macrophage senescence, inflammatory activation, and polarization. These rescue experiments are consistent with the involvement of DLG1/Kv1.3-related signaling in these phenotypes, although they do not establish a definitive causal pathway. We therefore propose that GPRC5B may be linked to a DLG1/Kv1.3-related signaling module; however, whether this relationship reflects transcriptional regulation, altered protein stability, ubiquitination-dependent degradation, or changes in protein localization remains to be determined. This mechanistic insight adds depth to the study because it links transcriptomic discovery to a defined downstream pathway.

The *in vivo* results further strengthen the biological and therapeutic relevance of this mechanism. GPRC5B knockdown in PMO mice was associated with an apparent trend toward improved bone structure and reduced macrophage senescence, suggesting that modulation of this pathway may have phenotypic consequences beyond cell culture models, although this remains to be confirmed by quantitative analysis. The observation that DLG1 inhibition further enhanced the protective effects of GPRC5B knockdown is particularly informative, because it supports the functional importance of the DLG1/Kv1.3 axis in the disease context. From a translational perspective, these findings raise the possibility that interfering with macrophage senescence pathways may represent an alternative or complementary therapeutic strategy in PMO. Rather than focusing only on osteoclast inhibition or bone anabolism, targeting disease-associated immune dysfunction may help restore a more favorable marrow environment for skeletal maintenance.

Our drug-prediction analysis provides an initial step in this direction. Doramectin was identified as a potential GPRC5B-targeting compound by DSigDB screening and molecular docking, and it reduced GPRC5B expression in estrogen-deprived macrophages. Although this evidence remains preliminary, it is encouraging because it suggests that GPRC5B may be pharmacologically tractable. At the same time, caution is warranted. Molecular docking and expression suppression do not by themselves establish *in vivo* efficacy or target specificity. Further studies will be needed to determine whether Doramectin directly binds GPRC5B in biological systems, whether it improves bone outcomes in PMO models, and whether its effects are mediated specifically through macrophage regulation. Nevertheless, identifying a candidate compound at this stage strengthens the translational relevance of the present work.

Several limitations should be acknowledged. First, the transcriptomic analyses were based on public datasets with limited sample sizes, heterogeneous clinical backgrounds, potential inter-cohort variability and expression profiling of circulating peripheral blood immune cells rather than bone marrow or bone tissue. Therefore, these findings should be interpreted as exploratory. Second, our *in vitro* systems modeled different aspects of PMO-related stress, including oxidative stress and estrogen deprivation, and therefore should not be interpreted as fully equivalent biological states. Third, macrophage senescence-associated changes were assessed mainly by SA-β-galactosidase staining together with inflammatory, polarization and phagocytic readouts. Because canonical senescence markers such as p16, p21, p53 and γH2AX were not examined, the current data do not definitively establish a complete cellular senescence feature. Future studies using multiple complementary senescence markers are required. Fourth, although our data support the involvement of GPRC5B and DLG1/Kv1.3 related signaling, the current loss-of-function evidence does not establish whether GPRC5B is sufficient to induce these phenotypes under basal conditions, and gain-of-function studies are required. Moreover, the precise regulatory mechanism linking GPRC5B to DLG1/Kv1.3 remains unclear, including possible effects on transcriptional regulation, protein stability, ubiquitination, and subcellular localization. Fifth, intra-bone marrow AAV-shGPRC5B delivery was not macrophage-specific, and effects on other bone marrow cell populations cannot be excluded. Future studies using macrophage-specific conditional knockout models or macrophage-targeted delivery approaches are required to determine the macrophage-specific *in vivo* function of GPRC5B. Sixth, the bone phenotypes were assessed only from representative micro-CT reconstructions, without quantitative trabecular parameters (bone volume fraction, trabecular thickness, trabecular number, trabecular separation) or BMD; standardized quantitative micro-CT analysis will therefore be needed in future work to confirm these apparent structural changes. Seventh, the DLG1 inhibitor was tested at a single dose without dose-response, pharmacokinetic, or target-engagement analyses, and thus the current data cannot establish pharmacological synergy between GPRC5B knockdown and DLG1 inhibition. Finally, the Doramectin-related findings should be regarded as preliminary and hypothesis-generating. Doramectin is a veterinary antiparasitic and was used only as a candidate compound for the druggability of GPRC5B. Direct binding, target specificity, *in vivo* efficacy, and the mechanism of GPRC5B mRNA reduction remain to be established.

## Conclusions

In summary, our study integrates single-cell, bulk transcriptomic, and experimental analyses to identify GPRC5B as a macrophage-associated candidate in PMO. The data support a model in which GPRC5B is linked to senescence-associated macrophage changes, altered inflammatory and phagocytic features, and bone phenotypes, potentially in connection with DLG1/Kv1.3-related signaling. Although these findings remain preliminary and require further mechanistic validation, they expand the current view of immune-associated alterations in PMO and provide a basis for future investigation of GPRC5B in osteoporosis-related bone marrow remodeling.

## Data Availability

The public datasets analyzed during the current study are available in the Gene Expression Omnibus (GEO) repository under accession numbers GSE147287, GSE169396, GSE56815, GSE7158 and GSE7429. Additional data supporting the findings of this study are available from the corresponding authors upon reasonable request.
