# Supplementary material for: Integrated single-cell and experimental analyses implicate GPRC5B in macrophage senescence-related alterations in postmenopausal osteoporosis
Source: Front Immunol. 2026 Aug 18;17:1890278. doi: 10.3389/fimmu.2026.1890278 (PMC13529568; doi:10.3389/fimmu.2026.1890278)
Supplement: Supplementary file 1 [file DataSheet1.docx]

**Integrated single-cell and experimental analyses implicate GPRC5B in macrophage** **senescence-related alterations in postmenopausal osteoporosis**

**Running title: GPRC5B in Macrophage Dysfunction of PMO**

**Supplementary Figure 1. Gene module analysis and core gene identification**

(A) Scale-free topology model fit plot showing the relationship between the soft-thresholding power and the scale-free topology model fit.

(B) Dendrogram of gene modules showing the hierarchical clustering of genes into modules.

(C) Bar plot showing eigengene-based connectivity (kME) for each gene module. Each subplot illustrates the distribution of kME values, and the genes enriched in each module are listed on the right, indicating the module-specific gene composition.

(D) Dot plot showing the average expression of genes across different macrophage subtypes in various modules. The color of the dots represents the average expression of genes, and the size of the dots indicates their expression percentage.

(E) Gene co-expression network of the pink module. Nodes represent genes, and edges represent co-expression relationships within the pink module. Each node is labeled with the corresponding gene name, illustrating the interconnectedness of genes in this specific co-expression module.

(F) Venn diagram showing the intersection of WGCNA-pink and KRAS pathway genes. The Venn diagram shows the overlap between genes in the pink module and genes up-regulated in the KRAS pathway.

**Supplementary Figure 2. Diagnostic performance and functional analysis of GPRC5B**

(A-C) ROC curves for GPRC5B in different datasets. The ROC curves show the diagnostic performance of GPRC5B in GSE56815 (A), GSE7158 (B), and GSE7429 (C) datasets. GSE56815, AUC = 0.812, 95% CI 0.677-0.947; GSE7158, AUC = 0.780, 95% CI 0.601-0.959; GSE7429, AUC = 0.700, 95% CI 0.466-0.934.

(D-F) GO enrichment analysis of GPRC5B-related functions in three datasets. The bar plots show the enriched biological processes related to GPRC5B, including antibacterial and immune responses (D), natural killer cell and lymphocyte-mediated immunity (E), and phagocytosis, immune signaling, and metabolic processes related biological processes (F). NES (Normalized Enrichment Score) and adjusted P-values are indicated, showing the significance and magnitude of enrichment. AUC values are shown with 95% confidence intervals estimated by the Hanley-McNeil method.

**Supplementary Figure 3. Immune infiltration and chemokine expression analysis of GPRC5B**

(A-C) Correlation heatmaps illustrating the association between GPRC5B expression and immune cell infiltration (including antigen-presenting cells, T cells, Macrophages, MHC molecules, inflammatory/para-inflammatory signatures, and IFN responses) across three datasets (GSE56815, GSE7158, and GSE7429). The color gradient represents the Pearson correlation coefficient (r), with red indicating positive correlation and blue indicating negative correlation.

(D) Correlation matrix showing the relationship between GPRC5B and chemokines in three datasets (GSE56815, GSE7158, GSE7429), with color indicating correlation strength (red for positive, green for negative) and asterisks denoting statistical significance (*p<0.05, **p<0.01, ***p<0.001).

(E) Correlation matrix showing the relationship between GPRC5B and TNF family in three datasets (GSE56815, GSE7158, GSE7429), with color indicating correlation strength (red for positive, green for negative) and asterisks denoting statistical significance (*p<0.05, **p<0.01, ***p<0.001).

**Supplementary Figure 4. Cell communication and metabolic pathway analysis of GPRC5B-positive macrophages**

(A) UMAP plot showing the distribution of GPRC5B expression in various cell types.

(B) Violin plot of GPRC5B expression in different immune and stromal cell types including Neutrophil, BM-MSC, RBC, B cell, Plasma cell, Macrophage, T cell, Endothelial cell, Monocyte, and Neutrophil progenitor. The violin shape represents the density of expression, and the central dot indicates the median expression.

(C) UMAP plot of GPRC5B expression in macrophage.

(D) Violin plot of GPRC5B expression in macrophage subtypes including Mac_APOE+, Mac_ADI1+, Mac_FTL+, Mac_CCL3+, Mac_SPP1+, and Mac_S100A8+. The violin shape reflects expression density, and the central dot denotes the median expression.

(E) Cell-cell interaction network involving GPRC5B-positive and negative populations. Nodes represent cell types, and edges represent the number of interactions between cell types. The size of nodes and the thickness of edges correspond to the number of interactions, illustrating the communication patterns centered around GPRC5B-expressing cells.

(F) The heatmap shows the communication probability (color gradient from blue to red) between GPRC5B-expressing cells and other cell types.
